# What is needed in culturally competent healthcare systems? A qualitative exploration of culturally diverse patients and professional interpreters in an Australian healthcare setting

**DOI:** 10.1186/s12889-019-7378-9

**Published:** 2019-08-13

**Authors:** Jennifer White, Trish Plompen, Leanne Tao, Emily Micallef, Terrence Haines

**Affiliations:** 1 School Primary and Allied Health Care, Monash University, Melbuorne, Victoria Australia; 2 Partnerships & Service Design, Monash Health, Melbourne, Victoria Australia; 3 School of Primary and Allied Health Care, Monash University, Moorooduc Hwy, Frankston, VIC 3199 Australia

**Keywords:** Access to care, Culture competence, Inpatient medicine, Interpreters, Qualitative

## Abstract

**Background:**

Culturally competent health care service delivery can improve health outcomes, increasing the efficiency of clinical staff, and greater patient satisfaction. We aimed to explore the experience of patients with limited English proficiency and professional interpreters in an acute hospital setting.

**Methods:**

In-depth interviews explored the experiences of four culturally and linguistically diverse communities with regards to their recent hospitalisation and access to interpreters. We also conducted focus group with professional interpreters working. Data were analysed using an inductive thematic approach with constant comparison.

**Results:**

Individual interviews were conducted with 12 patients from Greek, Chinese, Dari and Vietnamese backgrounds. Focus groups were conducted with 11 professional interpreters. Key themes emerged highlighting challenges to the delivery of health care due distress and lack of advocacy in patients. Interpreters struggled due to a reliance on family to act as interpreters and hospital staff proficiency in working with them.

**Conclusions:**

In an era of growing ethnic diversity this study confirms the complexity of providing a therapeutic relationships in contemporary health practice. This can be enhanced by training towards the effective use of professional interpreters in a hospital setting. Such efforts should be multidisciplinary and collective in order to ensure patients don’t fall through the gaps with regards to the provision of culturally competent care.

**Electronic supplementary material:**

The online version of this article (10.1186/s12889-019-7378-9) contains supplementary material, which is available to authorized users.

## Background

Australia has diverse and growing migrant populations with 190 different countries and 300 different ancestries represented [1]. Results from the most recent Australian Census showed that that up to 28.5% of the Australian population where born overseas 2016 [2]. It has been well established that patients from culturally and linguistically diverse backgrounds (CALD) experience poorer healthcare access and health care outcomes. These disparities have been shown to exist even when CALD population have similar medical conditions to English speaking patients [3, 4]. Such disparities are in part due to language barriers which make communication with the health system difficult [5]. How to provide accessible and quality health care is a serious challenge for the Australian health system.

Patterns of health care access among CALD patients with low English proficiency (LEP) highlight the experience of compromised care, longer hospital stays, higher rates of medical errors, and poorer patient satisfaction [6–9]. Existing health services need to adapt in order to promote the health and wellbeing of CALD populations. The provision of a culturally competent health care service has been posited as a key factor towards improving health outcomes, increasing the efficiency of clinical staff, and improving patient satisfaction. Growing work highlights key organisational components that have been shown to promote cultural competency including: the provision of health professional training and creating policies that streamline care and facilitate communication [10]. However barriers towards the provisions of culturally competent care include a lack of value in and resources towards staff training and education as well as the provision of culturally appropriate health education materials and other support to help patients navigate the health system [11].

Effective communication coupled with identification of and respect for cultural differences is essential to the delivery of culturally competent care. To achieve this health services need to facilitate clinician understanding of how patients from CALD populations understand disease and illness, view the causation and prognosis of their illness, describe their symptoms, understand treatment processes and how they perceive their role [12]. For example, some CALD patients do not consider it acceptable behaviour to ask questions within the health care context. As a results they are greater risk of not understanding their condition, being able to follow their treatment plan nor understand their health care rights [13, 14].

In an Australian context, interpreters are frequently provided as a means to overcome language barriers and help CALD populations to interact with and navigate the healthcare system. Despite evidence showing the association between interpreter access and better health care experiences and outcomes for patients with LEP [15–18], there remains an under-use of interpreters. Reasons for not using interpreters stems from the perceived lack of interpreter availability and increased workload when using interpreters [15, 19, 20]. Instead health services frequently use family members or untrained staff, such as bilingual or non-medical staff, to act as interpreters [21]. Use of non-accredited interpreters has been shown to compromise the quality of care as they lack vocabulary to interpret complex medical terminology, leading to misunderstanding and errors in translation toward key care components such as symptom identification and participation in medical decision making [21, 22]. It is important for us to understand the impact of not providing professional interpreter services on patient health care journeys. Findings can identify gaps in service provision that are not consistent with culturally competent health care and inform practice improvement.

As part of a larger study exploring hospital related outcomes of patients from culturally and linguistically diverse backgrounds [23], we aimed to explore the experience of patients with limited English proficiency and professional interpreters in an acute hospital setting.

## Methods

### Study design

This qualitative study explored the delivery of culturally competent health care in an Australian acute hospital setting. A data source triangulation approach was used to increase study validity and data richness by capturing different perspectives of the same phenomenon [24]. First, we undertook semi-structured interview across four culturally and linguistically diverse communities with regards to their recent hospitalisation and access to interpreters. We also conducted focus groups (FGs) with professional interpreters working at Monash Health. Recruitment occurred between May and June 2017. This project received approval from the Monash Health Human Research Ethics Committee (project number 14293Q).

### Recruitment

#### Patients

Potential participants were recruited as part of a larger study (methods reported elsewhere [23]). In brief, patients admitted to the General Medicine program at Monash Medical Centre (MMC) or Dandenong Hospital (DH) during the 2015–16 financial year, who were identified as having LEP and whose preferred language was either Greek, Chinese, Dari or Vietnamese. These languages were chosen as they were the most prevalent participant groups meeting the following criteria: admitted and received services from a trained interpreter on at least one occasion during an admission in the 2015–16 financial year with an additional admission in the same year without access to a trained interpreter were prioritised. Patients meeting inclusion criteria were mailed a letter of invite to the study, including study information, in their preferred language. After 2 weeks the researchers, using relevant interpreters, phoned each patient to answer questions and make an interview time at a convenient time and location, typically the patients home. Written consent was obtained prior to the interview commencing.

#### Interpreters

Focus groups were conducted at MMC (*n* = 6) and DH (*n* = 5). Recruitment was open to all professional interpreters, however to maximise participation the FGs were arranged by the Interpreter Service site manager at each site. Thus FGs formed a convenience sample. Detailed information was given about the study, time and location of each focus group and all participants provided written informed consent.

### Date generation

#### Patient

In-depth interviews (*n* = 12) were conducted by two researchers (JW and TP) with the assistance of interpreters using an interview schedule [25]. Interviews began by asking participants to share their ‘story’ of hospital admission and subsequent questions explored their experience of access to an interpreter or not. The semi-structured nature permitted flexibility for participants to elaborate upon or cover important topics that would not have otherwise surfaced, for example the experience of feeling confusion during ward rounds [26]. Emergent themes informed continuing data collection and sampling continued until thematic saturation (two co-coders agreeing that no new themes were emerging) was achieved.

#### Focus groups

Focus group methodology has been described elsewhere [23]. Questions specific to this study’s research aim explored the experience of interpreting for patients from culturally and linguistically diverse backgrounds in a busy, acute hospital setting. Thematic saturation was achieved.

### Analysis

Qualitative data analysis has been described in an earlier publication of clinician experience of language discordance [23]. Briefly, interview and FGs were recorded with participant consent and transcribed verbatim, with identifying data removed. Data analysis was guided by an inductive thematic approach [27] whereby the first step involved sustained engagement with the data through an initial reading and re-reading of transcripts to identify units of meaning and initial codes. Following team discussion initial codes were used to identify key categories and the primary author merged codes. In the final step, emerging categories were refined and grouped together into a theme. Rigour was upheld through immersion in data, reflexive analysis, peer debriefing and consensus coding between team members and discussion with a broader team [28]. Coders also captured exemplar quotes supporting each theme.

## Results

### Patient themes

Participant demographics are shown in Additional file 1: Table S1. Four distinct themes emerged from the Greek, Mandarin, Dari and Vietnamese participant interviews included in this study. These are discussed as follows.

1. *“I don’t want to complain…” – Limitations towards advocacy.*

Many participants reported experiencing emotional distress during their hospital admission. Feelings of worry and uncertainty were compounded by language barriers and not being able to communicate in a common language.

*“I feel over overwhelmed. I felt not able to communicate [in my own language] - this was very torture-some.” (Mandarin, Participant (P)1)*

Several participants reported that the assessment process was not clearly explained to them. Despite not understanding what was happening participants reportedly did not ask questions as they trusted that they were receiving appropriate care, being in a hospital setting that was considered a higher standard compared to their country of origin. Participant reports also suggested they were less likely to complain or advocate for their needs given their limited understanding of the Australian health system and awareness of their rights and responsibilities as a patient.

*“Because we are unfamiliar with the hospital protocol and procedure….so, we will just follow what the doctors’ instructions are.” (Mandarin, P2)*

Lack of self-advocacy was evident in most participants who relied on the hospital to arrange an interpreter for them. However few participants reported being offered an interpreter. Participants with some understanding of English were more confident to advocate for their own needs, including asking for an interpreter, and expressed concern for patients who had limited comprehension of English.

*“If you can’t communicate effectively I would feel a bit in a disadvantage position, but on the other hand I do understand a bit of it. There are other patients who don’t, and for them it’s even worse.” (Greek, P5).*

In fact the majority of participants assumed a passive role in communication with health professionals. For example participants reportedly listened to information provided to them during ward rounds and other medical consults, often not understanding or contributing to the conversation unless they were asked directly or prompted. This led to feeling “*left outside of the conversation.(Greek P2)”*

*“Yeah, there were times that the doctors were coming and saying the stuff and there was no interpreter and then I wouldn’t understand what they said.” (Dari P1)*

However feelings of being reassured by staff were reflected in participants’ expressions of confidence in the doctors, their competence and intention.

*“Whatever they offer I accept. I have no knowledge in the health area so I thank to the doctor and the nurse helping me.” (Vietnamese, P1)*

2. *“If there can be interpreters present - that will be the best thing” - Experiences of access to and use of interpreters.*

Access to interpreters reportedly varied among participants and there was consensus that greater access to interpreters would have been beneficial during admission.

*“I don’t want to create trouble with the hospital and also I have to face my language difficulty - if there can be interpreters present that will be the best thing.” (Mandarin, P3)*

In contrast all participants reported that the hospital organised an interpreter when consent for a procedure was required.

*“They would call an interpreter, not all the times, not from the beginning, but I did have an interpreter whenever I had to do scans.” (Dari, P1)*

Participants who had had previous admissions to hospital often noted that their need for an interpreter has already been established and an interpreter was automatically booked. Alternatively other participants who had had multiple admissions and wanted to ensure access to an interpreter said their children would phone ahead to, “*Arrange with the hospital for an interpreter.”* (*Greek, P3*)

Similarly, there was a common perception that interpreters were not available after hours. Therefore when presenting to the hospital after hours some participants indicated that it was important to bring a family member with them who could act as an interpreter.

*“It depends when we had the admission, whether it was during the day or during the night, if it is at night we didn’t have an interpreter….. So, I would take one of our children along with us.” (Greek P1)*

Several participants felt guilty about having limited English proficiency which made them reticent to request to be a burden on the health system and ask for an interpreter.

*“I feel very bad due to the fact that I have been in Australia for 60 years and I can’t speak English and answer a question put to me in the English language.” (Greek P2)*

3. *“I feel overwhelmed…” – Limited communication causes frustration and isolation.*

Participants who didn’t request an interpreter or indicate when they didn’t understand what was happening often suffered. This was in part because they didn’t want to be a burden to staff. For example one participant never complained about being in excessive pain and instead relied on seeing a nurse for pain medication at the scheduled time.

*“I knew when they would come around because they would give me medication, and if I was in pain I will endure it until they arrive.” (Greek, P2)*

Participants noted differences in their treatment by staff as a result of not speaking English and a times felt discriminated against when nurses didn’t talk to or approach them.

*“Nurses would go more often to the patients who spoke English” (Greek P4).*

However, interactions with staff and other patients who spoke their language greatly improved distress and feelings of isolation. This was most notable for participants with limited family or social support or when hospitalised for a prolonged period, such as for rehabilitation.

*“Later on there were nurses, either they can speak in Mandarin or in Cantonese who are on duty, but then when none of them are on duty, we are stuck, but we have to cope with it… you have to cope with it.” (Mandarin P3).*

Feelings of being “over looked” and not involved in decision making led to feelings of frustration.

*“They don’t pay enough attention. They assume that I am an idiot - not worth it.” (Greek, P3)*

Some participants reported distressing experiences exacerbated by communication misunderstandings. Confusion about levels of independence meant some participants felt that nursing staff were unsupportive or, *“refused to help”* (Mandarin, P2). Another participant felt she was, “*kicked out of hospital.” (Greek P4)* when her readiness for discharge was not explained.

4. *“If I don’t understand, I ask to stop as my daughter is coming” – The role of family members.*

All participants stated that family members were considered integral to their hospital experiences and decision making towards health care. For example if a doctor proposed a treatment regime or the need for a procedure then participants were likely to request that their family members be consulted. This had the potential to significantly delay procedures and length of stay when waiting for family to visit, or when an interpreter consult needed to be organised to facilitate discussion with family members.

*“If I don’t understand, I say no stop, my daughter coming”. (Greek P1)*

Most participants were aware that by relying on family (typically adult children) to be involved in decisions, that they were placing them under stressful situations due to the challenge of interpreting complex medical information and to ensure accurate information exchange.

*“My children speak fluent English, but they don’t speak fluent Greek, especially when it comes to medical terminology and they can’t explain properly….. So…even if they can understand anything, they can’t express everything you know the way an interpreter would”. (Greek P3)*

### Interpreter themes

Interpreter demographics are shown in Additional file 2: Table S2. Three key themes emerged which are discussed as follows

1. *Constraints to accurate interpreting: “We are here to facilitate communication”*

#### Delayed involvement

Interpreters reported they often felt conflicted and constrained by scenarios which they perceived went *“against hospital policies”* (MMC, Focus Group (FG)) that aimed to ensure inclusion and accurate information exchange. In the first instance interpreters cited that family, bilingual staff and non-professional staff (e.g. cleaners) were called upon being considered quicker and more convenient than booking their services. Even when they had been booked, interpreters noted that staff preferred to grasp earlier opportunities to start assessments when family were visiting.

*“If staff think the daughter is here, the cleaner is here, that will be quicker, easier” (DH, FG)*

*“Staff have booked an interpreter – but when the daughter comes in before me then they [staff] would prefer to use the daughter rather than wait for me the interpreter.” (MMC, FG)*

Further when an interpreter booking hadn’t been made, and they were observed to be present on a ward, then interpreters felt they were, *“grabbed by staff” (MMC, FG)* to provide immediate and spontaneous interpreting. While interpreters obliged where they could it wasn’t always possible since there were already booked for another patient, or for an outpatient clinic.

*“They [staff) ask for me to stay back so I can tell the patients or can ask the patients a few questions about caring for them. I try to. ”(MMC, FG)*

#### Family as interpreters

All interpreters expressed concern regarding the ability of family members, typically adult children, to accurately interpret in a stressful situation. Accurate interpretation of medical terminology was considered difficult and interpreters felt there were no procedures towards establishing the ability of family to accurately translate before proceeding with a consult. Despite clear hospital processes promoting access to professional interpreters it was readily noted that both staff and family frequently over-estimated the abilities of a family member to assist before involving a professional interpreter.

*“When clinicians are communicating with patients up in the ward, there is an assessment made about the patient’s English language proficiency ….and it is not necessarily the patient who is making that assessment. Often they [clinicians] incorrectly assume that the patient has a greater level of language proficiency. Even patients tend to overestimate their ability to communicate in English because they can go and pay for a newspaper.”(DH, FG)*

Some interpreting scenarios where reportedly more challenging than others, particularly those when family insisted on interpreting despite the presence or an interpreter and when family with-held information from the patient.

*“[Patients] children often do not have sufficient language skills on the one hand and also, in other cases, interpret half the truth.” (MMC, FG)*

Further when family members were present interpreters reported they were reliant on the treating clinician to advise them whether to stay or not, even though this contradicted policy.

“We need the cooperation from the professional to help to work with us. If the professional keeps going with the family members, which is easy…every conversation we are left out.”*(MMC, FG)*

Overall interpreters felt if they were able to stay for a consult then they could step in to ensure accurate information was relayed.

*“And when you have a family member they don’t tell it exact, it is hard. But if I am there and they don’t speak properly then I can jump in and said ‘No it is not.’ There are two things not telling the truth or misinterpreting information.” (DH, FG)*

Overall, interpreters observed the implications of delays in accessing their services such as the need to more accurately clarify symptoms and assist in complex family scenarios. Failure to involve their services in a timely fashion was perceived to be inefficient since it often required an initial assessment to be repeated or led to the risk re-admission.

*“And then you start from scratch and then you feel like oh my God! And the patient is already frustrated, not understanding what is happening. They want go home soon. and if they do go home…they will be back into hospital again because the picture of their health is wrong from the beginning.” (MMC, FG)*

2 *Working with clinicians*

#### Staff familiarity and confidence

All interpreters expressed that their ability to interpret accurately was compromised when treating clinicians failed to use their services or were unaware of, or failed to attend to cultural factors. These had the potential to impact on patient care. Recurring scenarios reportedly involved families not informing patients, especially parents, the truth about their illness and prognosis or speaking on their behalf. Interpreters were aware if this culturally factor, stemming from a families desire to support their parent and reduce fear and burden but had the potential to cause confusion.

*“[They don’t tell them] because they don’t want their loved one to know what’s exactly going on -it is a cultural thing.” (DH, FG)*

Another barrier to accurate interpretation occurred when interpreters perceived the information being provided to the patient by a clinician was too complicated for a patient’s level of insight and ability to comprehend. For example, interpreters felt that clinicians did not appreciate the complexity of the information being conveyed to patients towards medication or chronic disease management.

*“The thing is you can’t assume that they [patient] know the connection between going home, taking medication and staying here are the same thing. But, you know, if you get the background you explain a little bit more as an interpreter ….that helps…”(DH, FG)*

Interpreters also felt conflicted when they perceived that patients weren’t given chance to speak even if that wanted to. Interpreters readily observed this as *“very disheartening”* (MMC, FG) from the patients’ point of view

*“They [staff] are not allowing the opportunity to ask questions or to understand what is going on.” (MMC, FG)*

3 *Concern for accurate information exchange*

All interpreters valued maintaining a sense of professionalism in their role as interpreters both before, during and after the interpretation situation. This entailed providing a complete and accurate interpretation, remaining impartial, and ensuring confidentiality to “facilitate communication” (DH, FG). However, interpreters reported that it was difficult to carry out their role when there was a high turnover staff who lacked awareness of their service or how to work with them

*“Lack of awareness of the service. I think sometimes as well is that given the high turnover of staff, not everyone is then familiar with how to access to service.” (MMC, FG)*

Interpreters identified gaps in the skills of clinicians, especially those not experienced with working with patients with LEP and/or interpreters. Highly valued skills included active listening, cultural awareness, resect for family involvement, respect, and responding to the patients’ needs and wishes. Interpreters greatly appreciated it when a clinician contacted them to clarify and confirm the outcome of a clinical consult and any cultural aspects. Alternatively some interpreters were proactive and would ring the treating clinician following a consult. However the timing of this was difficult for them coordinate.

*“If the clinician calls me afterwards say what you think about you know, I said this this this that, you know what I mean. In the session I cannot say, ‘excuse me you [patient] are not telling the truth’… it is not our role.”(MMC, FG)*

*“It is very difficult and often after the consultation I am busy, I am going somewhere else when I am stopped by the professional to say: ‘Can you say anything you know about the meeting and so forth’” (DH, FG)*

## Discussion

This qualitative study involving interviews with patients with LEP and professional interpreters highlighted barriers to the delivery of a high quality, culturally competent health service. A central finding of this study was that patients with LEP felt overwhelmed and frustrated when unable to advocate for themselves and were not involved in decision about their own care. However patients felt guilty when they didn’t speak English which subsequently lead to them being passive in encounters with health professionals. Professional interpreters felt their services were not given priority and that family members were used to interpret in the first instance, despite the potential for inaccurate information exchange. Similarly professional interpreters observed the reliance on bilingual staff as a convenient alternative communication strategy.

All participants reported the reliance on family members and untrained staff, who were not skilled in interpreting medical terminology, was a barrier to accessing professional interpreters. This is consistent with previous research demonstrating that untrained staff do not have the skills in accurately translate medical terminology [29]. Further unskilled interpreters may not perceive cultural norms such as when CALD patients are perceived as being shy or show agreement with clinicians, even though they don’t actually understand what is being said [30].

This study also highlighted the experience of distress in patients when they perceived they were being overlooked by clinicians. Evidence highlights that dealing with emotions is complex and this is further compounded in CALD patients when language barriers exist and when clinicians do not respond empathically [31, 32]. We posit that the provision of culturally competent care requires educating clinicians to better detect the presence of psychological disturbance.

The delivery of culturally competent care is a challenge to many countries experiencing increasing numbers of migration and our findings echo this challenge, despite legislation to facilitate the availability of professional interpreters [5]. We identify key training components for clinicians working with CALD patient such as: developing cultural awareness, involving patients in communication and information sharing, and how to effective work alongside professional interpreters. Further, we suggest that training towards providing culturally competent care also should be provided on an ongoing basis in order to sustain benefits to patients [33] and reinforce clinician behaviour [34]. However given barriers to the use of professional interpreters ongoing efforts need to made by clinicians and administrative to address barriers to access and meet legislative requirements [35, 36].

### Strengths and limitations

We have previously reported on the outcomes of the clinician experience of language discordance [23]. This study generates important in-depth insight into the patient journey from a patient and interpreter perspective. Findings consistently highlight the need for greater access to and use of professional interpreters in order to provide the opportunity for communication, reassurance and earlier evaluation and treatment where necessary.

## Conclusion

As many countries experience growing cultural diversity there is need for the provision of enhanced care to patients from CALD background and access to interpreters. This can be promoted by training towards the effective use of professional interpreters in a hospital setting. Such efforts should be multidisciplinary and collective in order to ensure patients don’t fall through the gaps with regards to the provision of culturally competent care.

## Additional files

Additional file 1: **Table S1.** Patient Characteristics. (DOCX 34 kb)

Additional file 2: **Table S2.** Interpreter Characteristics. (DOCX 14 kb)

## Data Availability

The qualitative data used and/or analysed during the current study are available from the corresponding author on reasonable request.

## Abbreviations

DH: Dandenong Hospital
FG: Focus group
LEP: Low English proficiency
MMC: Monash Medical Centre
