# Supplementary figures and images for: What is needed in culturally competent healthcare systems? A qualitative exploration of culturally diverse patients and professional interpreters in an Australian healthcare setting
Source: BMC Public Health. 2019 Aug 13;19:1096. doi: 10.1186/s12889-019-7378-9 (PMC6693250; doi:10.1186/s12889-019-7378-9)

**Additional file 1: Table S1: Patient Characteristics**


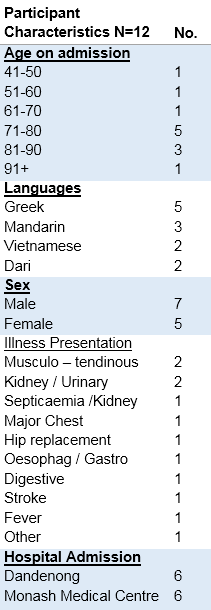

Supplement: Supplementary file 1 — Table S1. Patient Characteristics. (DOCX 34 kb) [file 12889_2019_7378_MOESM1_ESM.docx]
