# Supplementary material for: What is needed in culturally competent healthcare systems? A qualitative exploration of culturally diverse patients and professional interpreters in an Australian healthcare setting
Source: BMC Public Health. 2019 Aug 13;19:1096. doi: 10.1186/s12889-019-7378-9 (PMC6693250; doi:10.1186/s12889-019-7378-9)
Supplement: Supplementary file 2 — Table S2. Interpreter Characteristics. (DOCX 14 kb) [file 12889_2019_7378_MOESM2_ESM.docx]

**Additional file 2: Table S2: Interpreter Characteristics**

| Participant Characteristics 32 | No. |
| --- | --- |
| Age  26-30  31-35  36-40  46-50  51-55  56-60 | 3  2  2  1  2  1 |
| Educational Level  Bachelor Degree  Certificate/Diploma | 3  3 |
| Years in practice  1-4  5-9  10-14  15-19  >20 | 1  2  4  2  2 |
